# Pyoverdine Inhibitors and Gallium Nitrate Synergistically Affect Pseudomonas aeruginosa

**DOI:** 10.1128/mSphere.00401-21

**Published:** 2021-06-16

**Authors:** Donghoon Kang, Alexey V. Revtovich, Alexander E. Deyanov, Natalia V. Kirienko

**Affiliations:** a Department of BioSciences, Rice University, Houston, Texas, USA; Antimicrobial Development Specialists, LLC

**Keywords:** *Caenorhabditis elegans*, *Pseudomonas aeruginosa*, fluorocytosine, gallium, pyoverdine, tetracycline

## Abstract

Pseudomonas aeruginosa is a multidrug-resistant, opportunistic pathogen that frequently causes ventilator-associated pneumonia in intensive care units and chronic lung infections in cystic fibrosis patients. The rising prevalence of drug-resistant bacteria demands the exploration of new therapeutic avenues for treating P. aeruginosa infections. Perhaps the most thoroughly explored alternative is to use novel treatments to target pathogen virulence factors, like biofilm or toxin production. Gallium(III) nitrate is one such agent. It has been recognized for its ability to inhibit pathogen growth and biofilm formation in P. aeruginosa by disrupting bacterial iron homeostasis. However, irreversible sequestration by pyoverdine substantially limits its effectiveness. In this report, we show that disrupting pyoverdine production (genetically or chemically) potentiates the efficacy of gallium nitrate. Interestingly, we report that the pyoverdine inhibitor 5-fluorocytosine primarily functions as an antivirulent, even when it indirectly affects bacterial growth in the presence of gallium, and that low selective pressure for resistance occurs. We also demonstrate that the antibiotic tetracycline inhibits pyoverdine at concentrations below those required to prevent bacterial growth, and this activity allows it to synergize with gallium to inhibit bacterial growth and rescue Caenorhabditis elegans during P. aeruginosa pathogenesis.

**IMPORTANCE** P. aeruginosa is one of the most common causative agents for ventilator-associated pneumonia and nosocomial bacteremia and is a leading cause of death in patients with cystic fibrosis. Pandrug-resistant strains of P. aeruginosa are increasingly identified in clinical samples and show resistance to virtually all major classes of antibiotics, including aminoglycosides, cephalosporins, and carbapenems. Gallium(III) nitrate has received considerable attention as an antipseudomonal agent that inhibits P. aeruginosa growth and biofilm formation by disrupting bacterial iron homeostasis. This report demonstrates that biosynthetic inhibitors of pyoverdine, such as 5-fluorocytosine and tetracycline, synergize with gallium nitrate to inhibit P. aeruginosa growth and biofilm formation, rescuing C. elegans hosts during pathogenesis.

## INTRODUCTION

Pseudomonas aeruginosa is a Gram-negative, multidrug-resistant, opportunistic pathogen that threatens the lives of hospitalized patients, especially those in intensive care units. P. aeruginosa is one of the most common causes of ventilator-associated pneumonia (VAP) in these environments and has a high attributable mortality rate (1, 2). The importance of treating nosocomial VAP has become critical amid the coronavirus disease 2019 (COVID-19) pandemic, particularly since early studies have identified P. aeruginosa as one of the most common bacterial pathogens in COVID-19 patients (3, 4). P. aeruginosa also frequently infects patients who are immunocompromised due to cancer (5) and is the leading cause of chronic lung infections in patients with cystic fibrosis (6). Unfortunately, it is becoming increasingly difficult to treat P. aeruginosa infections due to the rising prevalence of drug-resistant strains. For example, our recent survey of multidrug-resistant P. aeruginosa isolates from pediatric patients with cystic fibrosis determined that a substantial fraction of the isolates were resistant to aminoglycosides, third- and fourth-generation cephalosporins, and even carbapenems, which are considered antibiotics of last resort for treating P. aeruginosa (7). The combination of increasing antibiotic resistance and the dwindling rate of new drug development is creating an urgent need for new therapeutics to treat these infections.

Recent work has bolstered the concept of targeting virulence determinants as an alternative treatment route. One common target is the siderophore pyoverdine, which is essential for bacterial growth under iron-restricted conditions, including during mammalian infections (8–11). Pyoverdine also regulates the production of secreted toxins such as the translational inhibitor exotoxin A and the protease PrpL (12). Interestingly, pyoverdine also disrupts host iron and mitochondrial homeostasis, even in the absence of the pathogen (13–16). A combination of these factors makes pyoverdine obligatory for P. aeruginosa virulence in murine lung infection models (9, 11, 17). Treatments that block pyoverdine biosynthesis (like the fluoropyrimidines 5-fluorocytosine or 5-fluorouridine) or pyoverdine function (like the small molecules LK11 or PQ3c) can substantially improve host survival under these conditions (7, 18–21).

Gallium(III) nitrate, Ga(NO_3_)_3_, has received considerable attention as an antipseudomonal therapeutic and has been shown to mitigate P. aeruginosa virulence in several murine infection models (22, 23). The widespread use of iron for redox biology across many metabolic pathways makes Ga(NO_3_)_3_ an effective antimicrobial (24). The most common explanation is that gallium(III) competes for binding sites in bacterial proteins and other molecules that are normally occupied by redox-active iron(III) (25). Since gallium(III) has an almost identical ionic radius but is redox inactive, its occupancy of these sites dramatically compromises their function.

In this report, we recapitulate previous findings that pyoverdine provides resistance to Ga(NO_3_)_3_ and extend them by showing that preventing pyoverdine biosynthesis potentiates gallium's antimicrobial activity. We show that the pyoverdine inhibitor 5-fluorocytosine synergizes with Ga(NO_3_)_3_ to inhibit P. aeruginosa growth and rescue Caenorhabditis elegans. We also demonstrate that this antivirulent maintains its low selective pressure for resistance even when it indirectly contributes to the inhibition of P. aeruginosa growth. Finally, we report that tetracycline-class antimicrobials attenuate pyoverdine production at concentrations lower than those that prevent bacterial growth, exhibiting synergistic interactions with gallium nitrate *in vitro* and *in vivo*.

## RESULTS

### Pyoverdine production confers gallium(III) nitrate resistance to P. aeruginosa.

**FIG 1:**
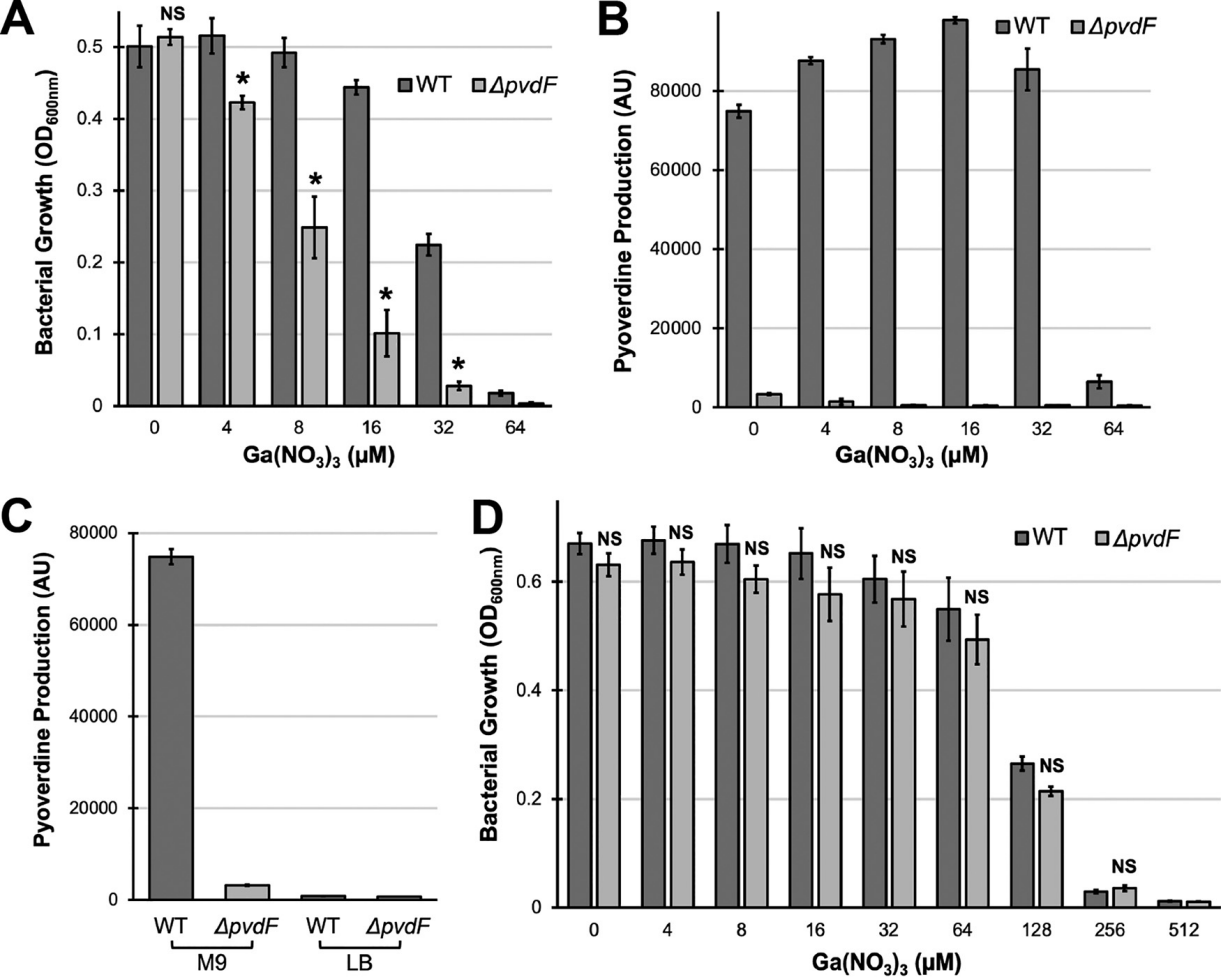
Pyoverdine production decreases P. aeruginosa susceptibility to Ga(NO_3_)_3_. (A and B) Bacterial growth (A) and pyoverdine production (B) by wild-type (WT) P. aeruginosa PAO1 and a pyoverdine biosynthetic mutant (PAO1*ΔpvdF*) in the presence of Ga(NO_3_)_3_ measured after 12 h incubation in M9 medium. (C) Pyoverdine production (in arbitrary units [AU]) by PAO1 and PAO1*ΔpvdF* in M9 and LB media. (D) Bacterial growth by PAO1 and PAO1*ΔpvdF* in the presence of Ga(NO_3_)_3_ in LB medium. Error bars represent standard errors of the mean (SEM) of three biological replicates. Statistical significance (Student’s *t* test) is indicated as follows: *, *P* < 0.01; NS, not significant (*P* > 0.05).

By virtue of being a ferric iron mimetic, gallium(III) is subject to chelation by the P. aeruginosa siderophores pyochelin and pyoverdine. Interestingly, this appears to have divergent effects depending upon which siderophore binds the metal. When pyochelin binds gallium(III), it deposits the metal into the cell where it interferes with cell function. Consequently, a pyochelin biosynthetic mutant, like P. aeruginosa Δ*pchBA* is more resistant to Ga(NO_3_)_3_ than wild-type P. aeruginosa (see Fig. S1 in the supplemental material). In contrast, pyoverdine appears to sequester gallium either outside the bacterium or within the periplasmic space (24, 26, 27), and P. aeruginosa Δ*pvdF*, which has compromised pyoverdine biosynthesis, is more susceptible to Ga(NO_3_)_3_ than wild-type P. aeruginosa in iron-limited media (Fig. 1A and B). This effect is abolished under conditions where pyoverdine is superfluous for survival, such as iron-rich media (Fig. 1C and D).

10.1128/mSphere.00401-21.1 FIG S1 Pyochelin production increases P. aeruginosa susceptibility to Ga(NO_3_)_3_. Bacterial growth (A and C) and pyoverdine production (B) by pyochelin biosynthetic mutant PAO1*ΔpchBA* in the presence of Ga(NO_3_)_3_ measured after 8 h incubation in M9 medium (A, B) or LB medium (C). Error bars represent SEM for three biological replicates. * corresponds to *P* < 0.01 and NS (not significant) corresponds to *P* > 0.05 based on Student’s *t* test. Download FIG S1, TIF file, 1.8 MB. Copyright © 2021 Kang et al. 2021 Kang et al. https://creativecommons.org/licenses/by/4.0/ This content is distributed under the terms of the Creative Commons Attribution 4.0 International license.

### 5-Fluorocytosine synergizes with gallium nitrate to inhibit P. aeruginosa growth and virulence.

**FIG 2:**
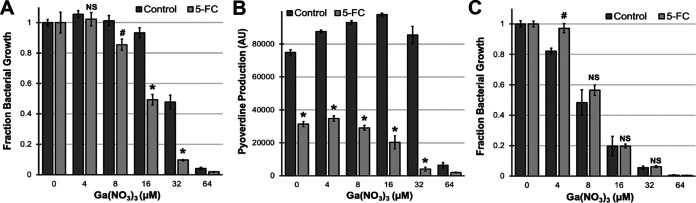
5-Fluorocytosine synergizes with Ga(NO_3_)_3_ to inhibit bacterial growth. (A to C) Bacterial growth (A) and pyoverdine production (B) by wild-type P. aeruginosa PAO1 or pyoverdine biosynthetic mutant PAO1*ΔpvdF* (C) in the presence of 100 μM 5-fluorocytosine (5-FC) and various concentrations of Ga(NO_3_)_3_ measured after 12 h incubation in M9 medium. Error bars represent SEM for four biological replicates. Statistical significance (Student’s *t* test) is indicated as follows: *, *P* < 0.01; #, *P* < 0.05; NS, not significant (*P* > 0.05).

Since pyoverdine production confers resistance to Ga(NO_3_)_3_, we predicted that compounds that prevent pyoverdine biosynthesis would potentiate the antimicrobial activity of gallium(III). Recent work has demonstrated that fluoropyrimidines (including 5-fluorocytosine, 5-fluorouridine, and 5-fluorouracil) inhibit pyoverdine production (18, 21). In particular, 5-fluorocytosine (5-FC) has been repeatedly shown to attenuate P. aeruginosa virulence during murine lung infection without exhibiting overt antibacterial activity *in vitro* (7, 18). We tested the interactions between 5-FC and Ga(NO_3_)_3_ (Fig. 2A). Bacteria were treated with a single concentration of 5-FC (100 μM), which substantially inhibits pyoverdine production but not bacterial growth (Fig. 2B), and a gradient of Ga(NO_3_)_3_. 5-FC increased the bacteriostatic activity of gallium(III) at concentrations as low as 8 μM (Fig. 2A). To eliminate alternative explanations, we performed this same test with an isogenic Δ*pvdF* mutant. 5-FC had no effect on gallium-mediated growth inhibition in the Δ*pvdF* mutant (Fig. 2C), demonstrating that this synergistic interaction is pyoverdine dependent.

**FIG 3:**
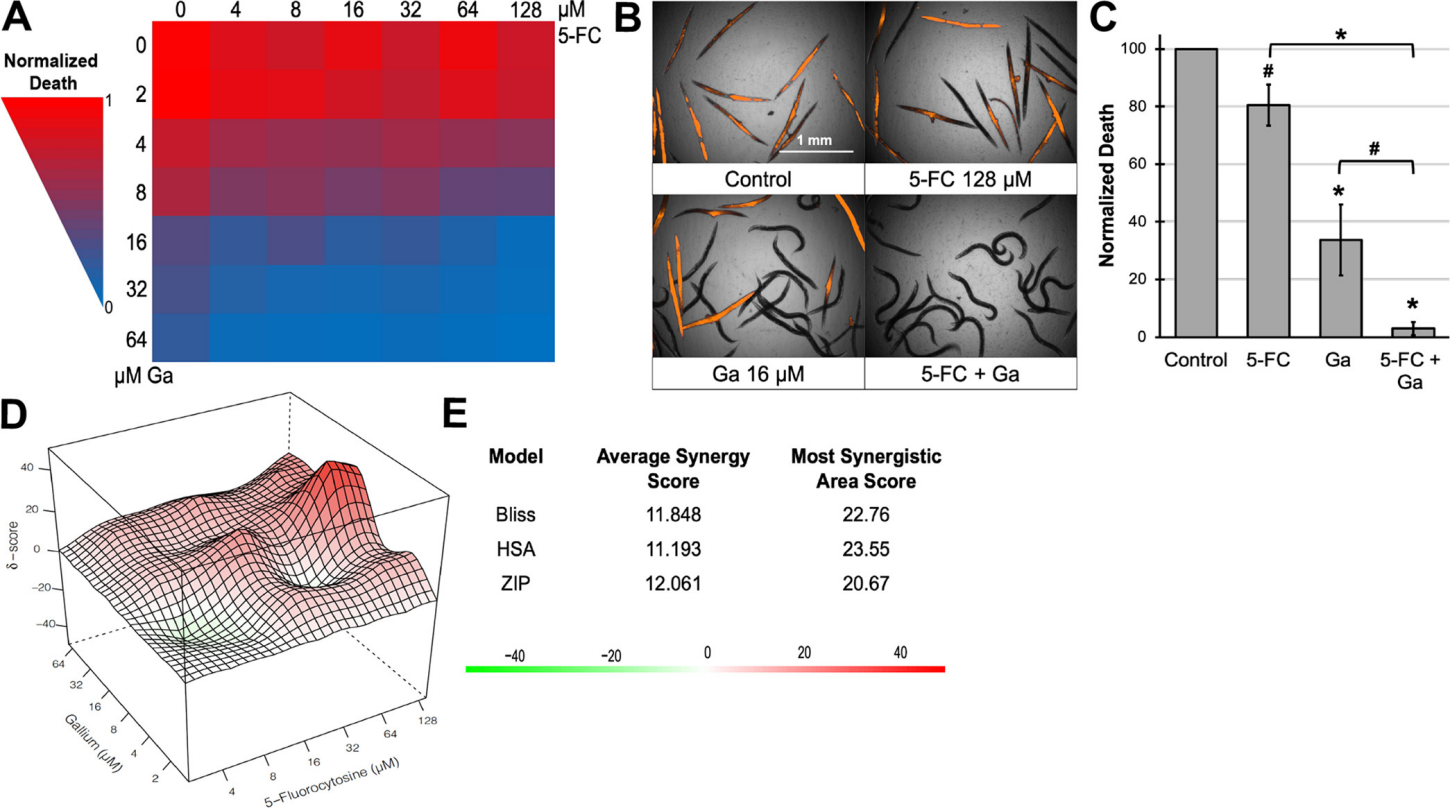
5-FC synergizes with Ga(NO_3_)_3_ to mitigate P. aeruginosa virulence. (A) Heatmap of normalized C. elegans death after exposure to P. aeruginosa in the presence of 5-fluorocytosine (5-FC) and Ga(NO_3_)_3_. Fraction host death was normalized to that of the no-drug control. (B) Fluorescent images of C. elegans stained with Sytox Orange cell impermeant nucleic acid stain. (C) Quantification of normalized C. elegans death. (D) 3-Dimensional synergy map for 5-FC and Ga(NO_3_)_3_ showing synergy scores (δ-score) for each drug combination. δ-scores were calculated based on the Bliss synergy model. (E) Average synergy scores and most synergistic area scores were calculated based on three different models. Error bars represent SEM for six biological replicates. Statistical significance (Student’s *t* test) is indicated as follows: *, *P* < 0.01; #, *P* < 0.05.

To investigate whether this *in vitro* synergy translates to the mitigation of bacterial virulence *in vivo*, we tested a range of drug combinations in a C. elegans pathogenesis model (14), where we previously showed that 5-FC rescues C. elegans in a pyoverdine-dependent manner (21). To explore the effects of gallium- and 5-FC-mediated growth inhibition in this model, we exposed worms to the pathogen for a longer period of time (∼65-h incubation compared to ∼42 h) to observe antivirulence (21). Under these conditions, 5-FC had minimal effect on pathogen virulence except at the highest concentration tested, 128 μM (Fig. 3A to C). It is important to note that this concentration is still physiologically relevant. We observed strong synergistic interactions between 5-FC and Ga(NO_3_)_3_ at several concentrations, where the drug combination resulted in near complete rescue of the host (Fig. 3B and C). To analyze these interactions in a broader context, we visualized the synergy scores for each drug combination using SynergyFinder (Fig. 3D) (28). For several drug synergy models, including the Bliss, highest single agency (HSA), and zero interaction potency (ZIP) models (29–31), we observed average synergy scores (δ-score) around 12, which corresponds to 12% greater effect than the expected outcome based on the performance of the individual drugs (Fig. 3E) (28). In the 3 × 3 concentration window where we observe the greatest synergy (most synergistic area), the combination of 5-FC and Ga(NO_3_)_3_ had an ∼22% greater effect on pathogen virulence than expected (Fig. 3E and Fig. S2), suggesting that this is a promising drug combination to attenuate P. aeruginosa pathogenesis.

10.1128/mSphere.00401-21.2 FIG S2 Interactions between 5-fluorocytosine and Ga(NO_3_)_3_ based on other synergy models. 3-Dimensional synergy maps for 5-fluorocytosine and Ga(NO_3_)_3_ based on their effect on P. aeruginosa virulence against C. elegans (heatmap shown in Fig. 3A). Synergy scores (δ) for each drug combination were calculated based on the Bliss (A), highest single agency (HSA) (B), or zero interaction potency (ZIP) (C) synergy models. Download FIG S2, TIF file, 2.3 MB. Copyright © 2021 Kang et al. 2021 Kang et al. https://creativecommons.org/licenses/by/4.0/ This content is distributed under the terms of the Creative Commons Attribution 4.0 International license.

### Pressure to develop resistance to 5-fluorocytosine remains low despite the presence of gallium nitrate.

One of the biggest motivations for the development of antivirulence therapeutics is their low selective pressure for resistance compared to conventional antimicrobials. Recently, Imperi and colleagues demonstrated that, while P. aeruginosa can become resistant to 5-FC through mutations in the uracil phosphoribosyltransferase (Upp) gene (32), the rate of resistance for 5-FC is orders of magnitude lower than for the analogous antibacterial compound 5-fluorouracil (33). Even after long-term exposure, 5-FC-resistant cells represented less than 0.1% of the population, which was insufficient to reduce the efficacy of pyoverdine inhibition (33).

**FIG 4:**
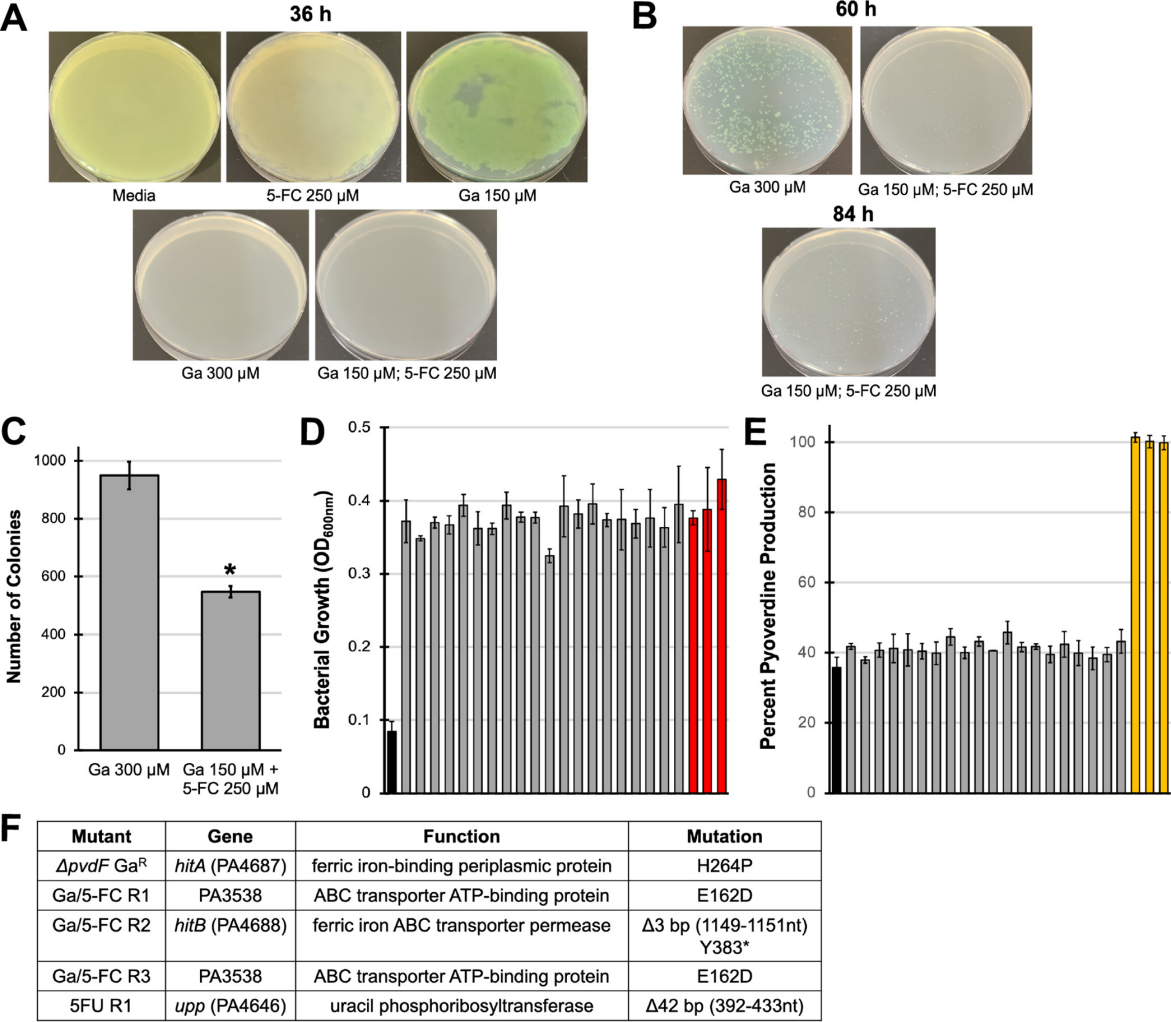
P. aeruginosa mutants adapted to Ga/5-FC remain sensitive to 5-FC. (A and B) Photographs of P. aeruginosa PAO1 grown on M9 agar plates supplemented with 5-fluorocytosine, Ga(NO_3_)_3_, or both after 36-h (A) or 60- to 84-h (B) incubation at 37°C. (C) Number of spontaneously resistant colonies counted after 60 h on 300 μM Ga(NO_3_)_3_ or 84 h on 150 μM Ga(NO_3_)_3_ and 250 μM 5-FC. (D) Bacterial growth in M9 medium supplemented with 64 μM Ga(NO_3_)_3_ for PAO1 parental strain (black), resistant colonies from the Ga/5-FC plate (gray), and resistant colonies from the 300 μM Ga(NO_3_)_3_ plate (red). (E) Percent pyoverdine production in M9 medium supplemented with 100 μM 5-FC for the PAO1 parental strain (black), resistant colonies from the Ga/5-FC plate (gray), and resistant colonies isolated from a plate containing 1 mM 5-fluorouracil (5-FU) (yellow). Pyoverdine production was normalized to that of the no-drug control. (F) Mutations found in various Ga(NO_3_)_3_ or 5-FU-resistant mutants. Error bars in panel C represent SEM for four biological replicates. Error bars in panels D and E represent SEM for two biological replicates. *, *P* < 0.01 by Student’s *t* test. nt, nucleotide.

We were interested in whether 5-FC’s indirect effect on bacterial growth in the presence of gallium would increase selective pressure for developing resistance. To adapt P. aeruginosa in the presence of the two drugs, we grew wild-type strain PAO1 on M9 agar plates containing 250 μM 5-FC and 150 μM Ga(NO_3_)_3_. For the first 36 h, we saw no discernible bacterial growth (Fig. 4A). After this time, we noticed the appearance of spontaneously resistant colonies (Fig. 4B). Consistent with the synergistic interactions observed in liquid media, M9 agar containing either one of these two drugs supported the formation of dense bacterial lawns (Fig. 4A). However, higher concentrations of Ga(NO_3_)_3_ (≥300 μM) were able to suppress bacterial growth (Fig. 4A). The presence of 300 μM gallium triggered the spontaneous emergence of resistant cells (Fig. 4B). At the same time, the combination of Ga(NO_3_)_3_ and 5-FC, even at a lower concentration of gallium, resulted in approximately half as many resistant colonies (Fig. 4C). The rate of colony growth was substantially lower on the Ga/5-FC plate (Fig. 4B), likely due to the pathogen’s inability to produce pyoverdine to alleviate gallium toxicity. From this plate, we isolated 40 individual colonies and passaged them through drug-free, nutrient-rich medium for further characterization.

Bacteria from all 40 colonies grew better in the presence of gallium than the parental strain (Fig. 4D and Fig. S3A), indicating that mutants have bona fide resistance and are not merely persisters. Interestingly, all mutants remained sensitive to 5-FC (Fig. 4E and Fig. S3B). On the other hand, spontaneously resistant colonies isolated from P. aeruginosa grown in the presence of 5-fluorouracil (5-FU) were resistant to 5-FC-mediated pyoverdine inhibition (Fig. 4E), which is consistent with our observations that 5-FC is likely to be metabolized through 5-FU to have its effect (18, 21). These results suggest that the effect of 5-FC is through its role as an antivirulent, even in the presence of gallium. This makes it more likely that treatment will continue to exert low selective pressure for evolving resistance.

10.1128/mSphere.00401-21.3 FIG S3 Phenotypes of P. aeruginosa mutants adapted to Ga(NO_3_)_3_ and 5-fluorocytosine. (A) Bacterial growth in M9 media supplemented with 64 μM Ga(NO_3_)_3_ for PAO1 parental strain (black), resistant colonies from the Ga/5-FC plate (gray), and resistant colonies from the 300 μM Ga(NO_3_)_3_ plate (red). (B and C) Percent pyoverdine production in M9 media supplemented with 100 μM 5-FC for the PAO1 parental strain (black), resistant colonies from the Ga/5-FC plate (gray), and resistant colonies isolated from a plate containing 1 mM 5-fluorouracil (5-FU) (yellow) (B). Pyoverdine production was normalized to that of the no-drug control (C). Error bars represent SEM for two biological replicates. Download FIG S3, TIF file, 1.9 MB. Copyright © 2021 Kang et al. 2021 Kang et al. https://creativecommons.org/licenses/by/4.0/ This content is distributed under the terms of the Creative Commons Attribution 4.0 International license.

### Mechanism of gallium nitrate resistance.

To elucidate the mechanism of gallium resistance in the Ga/5-FC-adapted mutants, we first investigated whether increased pyoverdine production decreased sensitivity to gallium. Interestingly, none of the 40 mutants exhibited increased pyoverdine production (Fig. S3C), suggesting that the mechanism of resistance was independent of this siderophore. Previous observations by Garcia-Contreras and colleagues indicated that increased pyocyanin production could confer resistance to gallium (34), so we measured that as well. Neither the parental strain nor representative mutants produced detectable levels of pyocyanin in M9 media (data not shown).

To study pyoverdine-independent resistance to gallium(III), a P. aeruginosa Δ*pvdF* mutant was plated on M9 agar containing 100 μM Ga(NO_3_)_3_. Like its wild-type counterpart, we noticed the appearance of spontaneously resistant colonies (Fig. S4A), which remained resistant to gallium after passaging in drug-free medium (Fig. S4B and C). We subjected one of the mutant strains to whole-genome sequencing and discovered a single mutation, which was located in the *hitA* gene (791A→C; H264P) (Fig. 4F). *hitA* encodes a periplasmic ferric iron-binding protein belonging to the HitABC class of transporters that is responsible for the delivery of ferric iron from the periplasm to the cytoplasm (35). Mutations in *hitA* and *hitB* have previously been shown to confer resistance to Ga(NO_3_)_3_ in a transposon mutagenesis screen (34).

10.1128/mSphere.00401-21.4 FIG S4 Spontaneous gallium resistance in a pyoverdine biosynthetic mutant. (A) Photograph of PAO1*ΔpvdF* spontaneous gallium-resistant colonies from bacteria grown on M9 agar plate with 100 μM Ga(NO_3_)_3_. (B and C) Bacterial growth by the PAO1*ΔpvdF* parental strain and gallium-resistant mutant in the presence of Ga(NO_3_)_3_ in M9 (B) or LB (C) media. Error bars represent SEM for three biological replicates. * corresponds to *P* < 0.01, # corresponds to *P* < 0.05, and NS (not significant) corresponds to *P* > 0.05 based on Student’s *t* test. Download FIG S4, TIF file, 2.3 MB. Copyright © 2021 Kang et al. 2021 Kang et al. https://creativecommons.org/licenses/by/4.0/ This content is distributed under the terms of the Creative Commons Attribution 4.0 International license.

We sequenced the genomes of three Ga/5-FC-adapted mutants to determine whether they carried similar mutations. Each mutant exhibited monogenic mutations: one mutant had a nonsense mutation in *hitB*, while the other two mutants carried the same glutamic acid-to-aspartic acid substitution (E162D) in the PA3538 gene (Fig. 4F). As Guo and colleagues demonstrated that PA3538, in combination with *hitAB*, allows the transport of both iron(III) and gallium(III) into E. coli, which normally lacks this transport function (35), it is likely that PA3538 represents the missing HitC protein for *P. aeruginosa*. Interestingly, none of these mutants carried mutations in *upp*, while the 5-FU-adapted strain exhibited a 42-bp deletion in this gene (Fig. 4F).

### Tetracyclines inhibit pyoverdine production.

**FIG 5:**
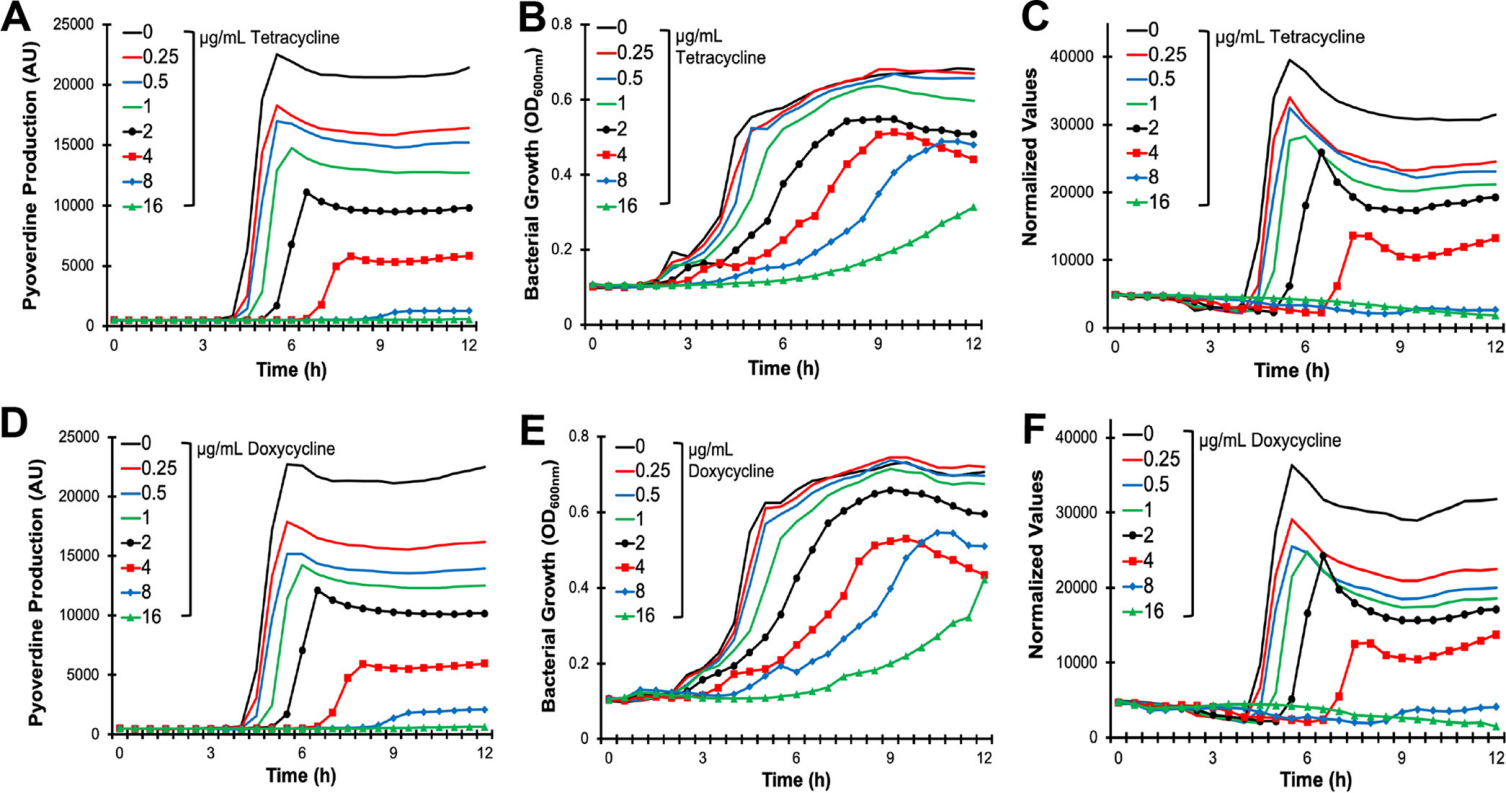
Tetracycline-class antibiotics inhibit pyoverdine production. (A to C) Bacterial growth (A), pyoverdine production (B), and pyoverdine production normalized to bacterial growth (C) of P. aeruginosa PAO1 in the presence of tetracycline. (D to F) Bacterial growth (D), pyoverdine production (E), and pyoverdine production normalized to bacterial growth (F) of P. aeruginosa PAO1 in the presence of doxycycline.

To test whether Ga(NO_3_)_3_ synergizes with other pyoverdine inhibitors, we turned to bacterial translational inhibitors. Pyoverdine biosynthesis requires at least 14 enzymes, including 3 large peptide synthetases, PvdL, PvdD, and PvdJ, that produce the 8- to 11-amino-acid side chain attached to the dihydroxyquinoline core (36). The large size of these proteins (each is greater than 2,000 amino acids) is likely to make them more sensitive to translational inhibition. We predicted that tetracycline-class antibiotics, which inhibit the 30S ribosomal subunit, would limit pyoverdine production, even at concentrations insufficient to compromise growth. We tested this by inoculating wild-type P. aeruginosa into media containing various concentrations of tetracycline (Fig. 5A to C) or doxycycline (Fig. 5D to F) and measured bacterial growth and pyoverdine production. We observed a decrease in pyoverdine production, even at concentrations where bacterial growth was unaffected. Higher (but still clinically relevant) concentrations of tetracycline-class antibiotics exhibited a strong bacteriostatic effect against P. aeruginosa and nearly abolished pyoverdine production.

**FIG 6:**
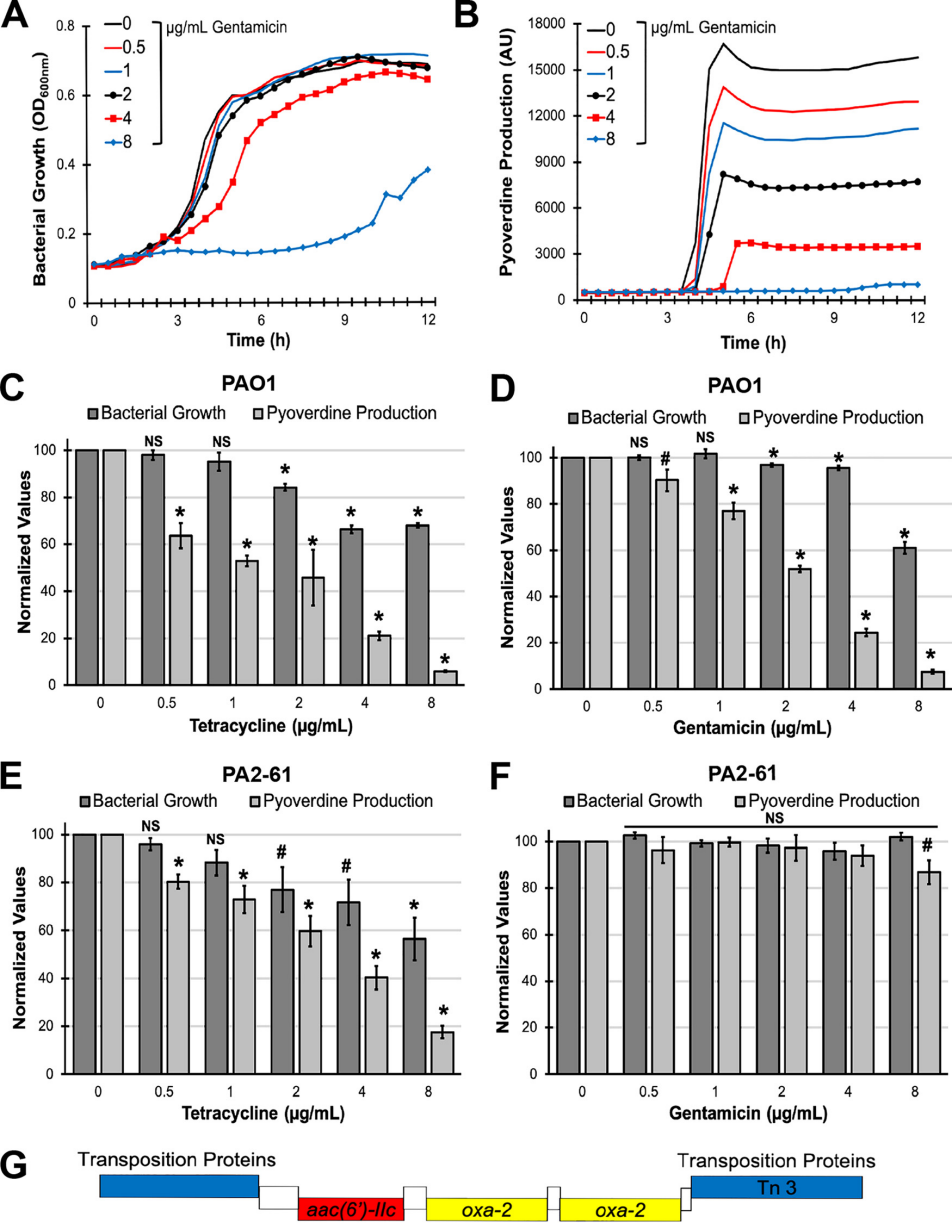
Gentamicin inhibits pyoverdine production only in drug-susceptible strains of P. aeruginosa. (A and B) Bacterial growth (A) and pyoverdine production (B) of P. aeruginosa PAO1 in the presence of gentamicin. (C to F) Relative bacterial growth and pyoverdine production of P. aeruginosa PAO1 (C and D) or cystic fibrosis isolate PA2-61 (E and F) in the presence of tetracycline or gentamicin measured after 12 h of incubation in M9 medium. Values were normalized to those of the no-drug control. (G) Diagram of the multidrug resistance transposon found in PA2-61. The *aac(6*′*)-IIc* gene expresses an aminoglycoside acetyltransferase conferring resistance to gentamicin. *oxa-2* encodes a beta-lactamase. Error bars represent SEM for three biological replicates. Statistical significance (Student’s *t* test) is indicated as follows: *, *P* < 0.01; #, *P* < 0.05; NS, not significant (*P* > 0.05).

We tested whether this was also true for gentamicin, an aminoglycoside antibiotic that also inhibits the 30S ribosomal subunit. As expected, gentamicin significantly curtailed pyoverdine production at subinhibitory concentrations (Fig. 6A to D). We extended this finding using P. aeruginosa PA2-61, a tetracycline- and gentamicin-resistant isolate from a pediatric cystic fibrosis patient (7). In this isolate, tetracycline maintained its bacteriostatic and pyoverdine-inhibitory activity, while gentamicin had no effect on bacterial growth or pyoverdine production (Fig. 6E and F). Consistent with this, we identified a multidrug resistance transposon in the genome of PA2-61 that encodes the aminoglycoside acetyltransferase AAC(6′)-IIc, which confers resistance to gentamicin, and two copies of an OXA-2 beta-lactamase (Fig. 6G). For cystic fibrosis isolates susceptible to gentamicin but resistant to tetracycline (PA2-72 and PA3-29) (7), both drugs substantially curtailed bacterial growth and pyoverdine production (Fig. S5).

10.1128/mSphere.00401-21.5 FIG S5 Tetracycline inhibits pyoverdine production in drug-resistant cystic fibrosis isolates. Relative bacterial growth and pyoverdine production by P. aeruginosa cystic fibrosis isolates PA2-72 or PA3-29 in the presence of tetracycline (A and C) or gentamicin (B and D) measured after 12 h incubation in M9 media. Values were normalized to those of drug-free controls. Error bars represent SEM for three biological replicates. * corresponds to *P* < 0.01, # corresponds to *P* < 0.05, and NS corresponds to not significant (*P* > 0.05) based on Student’s *t* test. Download FIG S5, TIF file, 1.0 MB. Copyright © 2021 Kang et al. 2021 Kang et al. https://creativecommons.org/licenses/by/4.0/ This content is distributed under the terms of the Creative Commons Attribution 4.0 International license.

One explanation for the discrepancy between the effects of tetracycline and gentamicin may arise from common mechanisms that provide resistance to these antibiotics. Tetracycline resistance in P. aeruginosa primarily occurs through the activity of multidrug efflux pumps (37, 38), while bacteria often biochemically modify aminoglycosides to render them inactive (39). Some P. aeruginosa strains carry versions of the tetracycline-inactivating enzyme *tet*(X) from Bacterioides fragilis (40, 41), which is a potential alternative explanation. We consider this less probable, however, as possession of a tetracycline-inactivating enzyme remains a rare, but currently emerging, phenotype in *P. aeruginosa* (42). Of these two mechanisms, inactivation of antibiotics is more likely to restore translational activity because it permanently reduces the concentration of the compound, rather than simply reducing intracellular concentrations. This allows tetracycline to continue to inhibit pyoverdine production. For these reasons, in subsequent experiments, our efforts focused on tetracycline as a better pyoverdine inhibitor than gentamicin.

### Tetracycline synergizes with gallium nitrate to inhibit P. aeruginosa.

**FIG 7:**
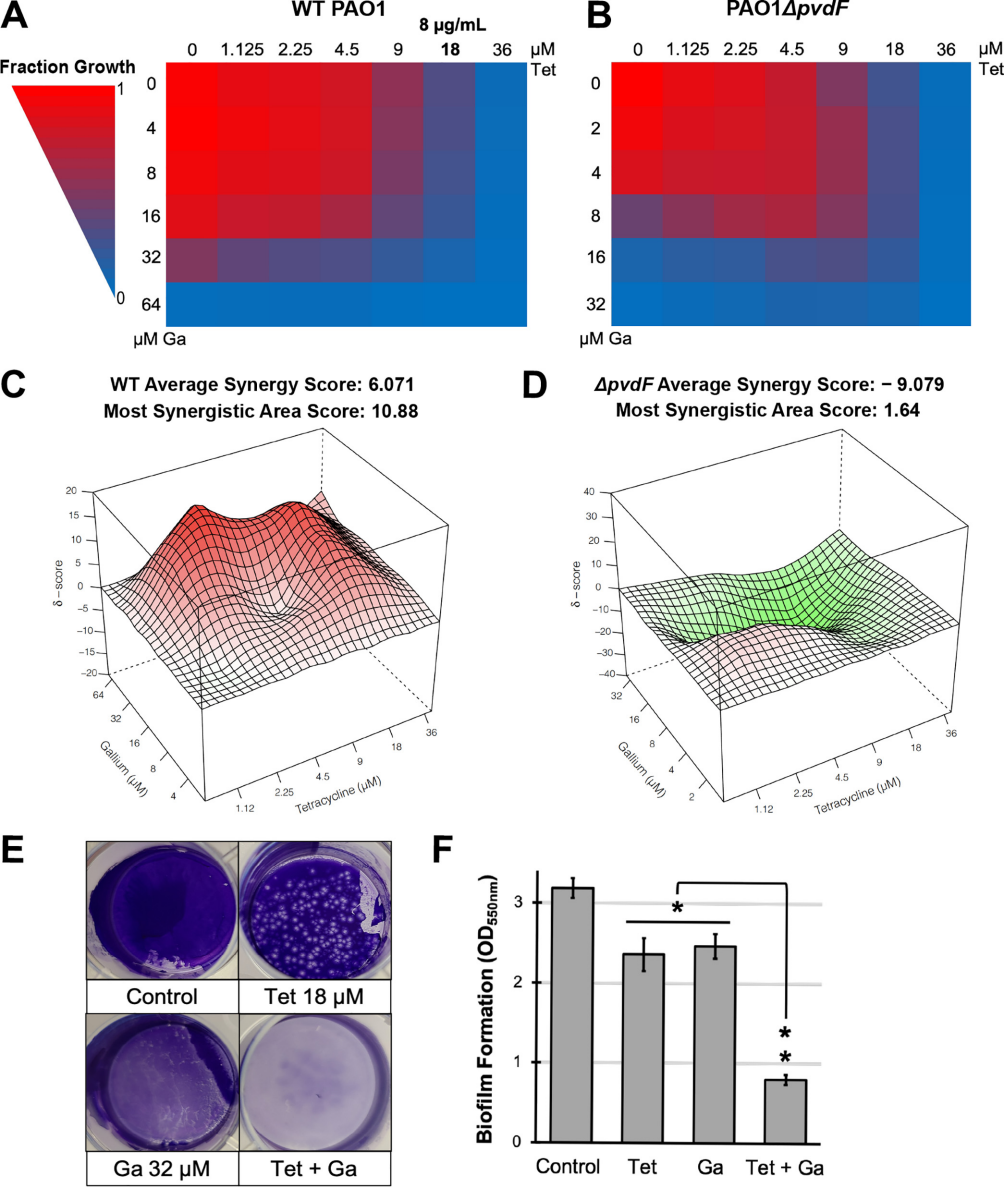
Tetracycline synergistically potentiates Ga(NO_3_)_3_. (A and B) Heatmap of PAO1 (A) or PAO1*ΔpvdF* (B) fraction bacterial growth in the presence of specified concentrations of tetracycline (Tet) and Ga(NO_3_)_3_ measured after 12 h of incubation in M9 medium. Values were normalized to those of the no-drug control. (C and D) 3-Dimensional synergy maps for tetracycline and Ga(NO_3_)_3_ showing synergy scores (δ-score) for each drug combination for wild-type PAO1 (C) and PAO1*ΔpvdF* (D). δ-scores were calculated based on the Bliss synergy model. (E) Photograph of P. aeruginosa biofilms stained with crystal violet after 20-h growth in M9 medium. (F) Quantification of crystal violet staining after acetic acid solubilization. Error bars represent SEM for three biological replicates. *, *P* < 0.01 by Student’s *t* test.

Unlike 5-FC, tetracycline exhibits antimicrobial activity, so we tested a gradient of concentrations of both tetracycline and Ga(NO_3_)_3_ (Fig. 7A). The combination of 18 μM (8 μg/ml) tetracycline and 32 μM gallium was able to effectively curtail P. aeruginosa growth (Fig. 7A). We used SynergyFinder (28) to more accurately evaluate the interactions between tetracycline and gallium. We observed modest, concentration-specific, synergistic interactions between the two drugs in wild-type P. aeruginosa (δ-score > 5) with the combination performing on average ∼6% better than the expected outcome based on the Bliss synergy model. While this average score was below the customary cutoff for strong synergy (δ-score > 10), the drug combination narrowly exceeded this standard in the area of maximal synergy (Fig. 7C). We observed similar results for the highest single agency and zero interaction potency synergy models (Fig. S6). As anticipated, no synergy was seen in the pyoverdine biosynthetic mutant, where we observed generally antagonistic interactions between the two compounds (Fig. 7B and D and Fig. S6).

10.1128/mSphere.00401-21.6 FIG S6 Interactions between tetracycline and Ga(NO_3_)_3_ based on other synergy models. 3-Dimensional synergy maps for tetracycline and Ga(NO_3_)_3_ based on their effect on bacterial growth for wild-type P. aeruginosa PAO1 (A, C, and E) or PAO1*ΔpvdF* (B, D, and F) (heatmaps shown in Fig. 7A and B). Synergy scores (δ) for each drug combination were calculated based on the Bliss (A and B), highest single agency (HSA) (C and D), or zero interaction potency (ZIP) (E and F) synergy models. (G) Average synergy scores and most synergistic area scores calculated based on three different models. Download FIG S6, TIF file, 1.5 MB. Copyright © 2021 Kang et al. 2021 Kang et al. https://creativecommons.org/licenses/by/4.0/ This content is distributed under the terms of the Creative Commons Attribution 4.0 International license.

**FIG 8:**
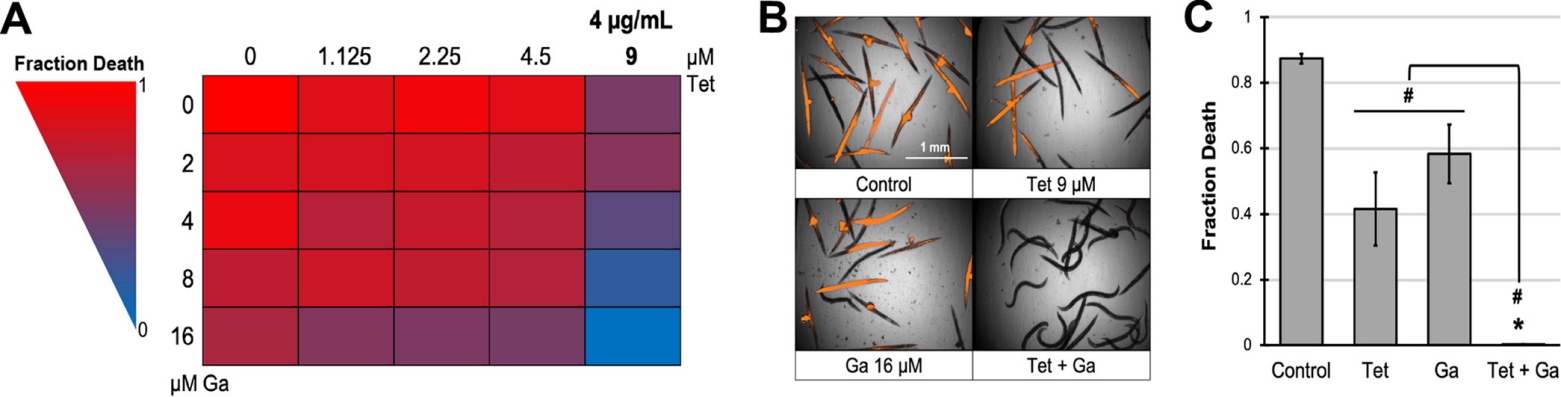
Tetracycline and gallium effectively mitigate P. aeruginosa virulence against C. elegans. (A) Heatmap of fraction C. elegans death after exposure to P. aeruginosa in the presence of tetracycline and Ga(NO_3_)_3_. (B) Fluorescent images of C. elegans stained with Sytox Orange cell impermeant nucleic acid stain. (C) Quantification of fraction C. elegans death. Error bars represent SEM for three biological replicates. Statistical significance (Student’s *t* test) is indicated as follows: *, *P* < 0.01; #, *P* < 0.05.

It is a well-established paradigm that pyoverdine supports biofilm formation by facilitating iron acquisition (43, 44), so we also tested whether the combination of tetracycline and gallium(III) was able to effectively inhibit P. aeruginosa biofilm formation. As anticipated, the combination strongly compromised biofilm development (Fig. 7E and F). Finally, we tested this drug combination in a C. elegans pathogenesis model. While tetracycline and Ga(NO_3_)_3_ exhibited a modest attenuation of virulence on their own, the combination of the two provided essentially complete rescue (Fig. 8).

## DISCUSSION

The rise of antimicrobial resistance is universally recognized as a critical threat to humanity. Despite this looming danger, antimicrobial development has waned, receiving little support from disinterested pharmaceutical programs or governments with other legislative priorities. The most effective governmental programs that have arisen involve stewardship and aim to stem further development of drug resistance by restricting the use of particular antibiotics. Unfortunately, these programs will not lead to the development of new treatments, and their efforts are stymied by regulatory apathy in other nations.

One popular alternative to the development of completely new chemical entities is to repurpose or reposition drugs that are already approved for other uses (45–47). For example, we recently reported that cancer chemotherapeutics (21) and the insulin mimetic demethylasterriquinone B1 (48) have some potential for being repurposed as antimicrobials. Since the biological characteristics are already known for compounds approved by the U.S. FDA, preliminary safety studies are much simpler, and scientists can move directly into efficacy studies.

Because gallium(III) is already FDA approved for some indications, it has been explored for repurposing as an antipseudomonal agent. This led to discoveries that gallium effectively treats P. aeruginosa infections in mice (22, 23). However, several obstacles limit the potential of this treatment, such as the relatively straightforward acquisition of resistance via mutation of *hitAB* (a ferric iron transporter), loss of pyochelin [which is involved in gallium(III) import], or the development of gallium efflux activity (26, 34, 49). Pyoverdine production is also known to decrease the efficacy of gallium(III) treatment (24), as we demonstrated here as well. This is likely to be a consequence of irreversible sequestration of the metal by pyoverdine. While both pyoverdine-gallium and ferripyoverdine translocate into the bacterium, pyoverdine-gallium accumulates in the periplasm (27). In the periplasm, iron(III) is reduced, lowering pyoverdine’s affinity and allowing the ferrous iron to be transported into the cytoplasm and the pyoverdine to be exported for reuse. Since gallium is redox inactive, the affinity is unchanged, and the metal cannot be released. Pyoverdine-gallium is also less efficiently translocated into the cell than ferripyoverdine (50) though similar observations were also made for pyochelin-gallium (51). The difference between the two siderophores is likely due to their affinities toward ferric iron and gallium. Pyochelin exhibits lower affinity toward ferric iron and is presumed to release the metal in its oxidized state, allowing pyochelin to function as a gallium shuttle rather than as a sink. On the basis of these principles, Frangipani and colleagues have demonstrated that the pyochelin-gallium complex is actually a more effective growth inhibitor than gallium nitrate alone (26). Similar observations have been previously made for the pyochelin-vanadium complex (52).

Another potential method to improve gallium(III) effect is to combine it with a conventional antimicrobial. This approach has also been documented, as Goss and colleagues investigated the interactions between gallium nitrate and a panel of antipseudomonal antibiotics. Gallium synergized with colistin and piperacillin-tazobactam but had no effect on the inhibitory effects of ceftazidime, ciprofloxacin, or aztreonam (22). Most notable, however, was that gallium exhibited antagonism with tobramycin, partially restoring bacterial growth inhibited by the antibiotic (22). This finding challenges the potential of gallium(III) as an antipseudomonal agent since tobramycin is currently the standard of care drug for inhaled antibiotic therapy in cystic fibrosis patients (53). Thus, the identification of additional therapeutics that exhibit either synergistic or additive interactions with gallium would be crucial for its clinical applicability. For instance, Halwani and colleagues demonstrated that the liposomal delivery of gallium and gentamicin is substantially more effective in inhibiting P. aeruginosa than the free drug treatment, even in a highly resistant clinical isolate (54).

In this report, we have demonstrated that pyoverdine inhibitors may be useful in combination with gallium(III). 5-Fluorocytosine (5-FC) and tetracycline synergized with gallium(III) to inhibit bacterial growth *in vitro* and mitigate P. aeruginosa virulence *in vivo*. Antivirulents such as 5-FC are considered a promising new class of therapeutics due to their presumably low selective pressure for resistance compared to conventional antimicrobials (55). However, this advantage remains controversial since infection conditions can provide selective pressure for pathogens to develop resistance against these drugs and become more pathogenic. This is likely the case for 5-FC because the molecule reduces pyoverdine production, interfering with iron uptake. P. aeruginosa can also easily acquire resistance through mutations in *upp*, its uracil phosphoribosyltransferase (32). However, we observed low pressure for resistance to 5-FC in the presence of gallium, even when 5-FC indirectly contributes to growth inhibition, suggesting that therapeutics that do not directly and overtly exhibit antibacterial activity may indeed have a longer shelf life than antibiotics.

However, it is also important to note that the synergy between gallium(III) and 5-FC or tetracycline depends on effectively limiting pyoverdine production. Unfortunately, P. aeruginosa clinical isolates constitute a highly diverse set of strains with often heterogeneous phenotypes. For instance, we recently reported that approximately one-third of P. aeruginosa cystic fibrosis isolates we tested from pediatric cystic fibrosis patients lost the ability to produce pyoverdine (7). Martin and colleagues have made similar observations from cystic fibrosis patient sputum samples (56). Others have taken this a step farther and demonstrated that P. aeruginosa adapts its iron acquisition strategy within the cystic fibrosis lung by transitioning from pyoverdine-mediated ferric iron uptake toward heme assimilation/utilization (57, 58).

Nevertheless, pyoverdine remains a critical acute virulence factor in a majority of P. aeruginosa clinical isolates. Pyoverdine production can also be rapidly measured from bacterial cultures or directly detected from patient samples using spectrophotometric tools due to its distinct spectral properties (56), allowing personalized pyoverdine-based treatments to be used. Identifying additional pyoverdine inhibitors (e.g., fluoropyrimidines, twin arginine translocase inhibitors [59, 60], quorum-sensing inhibitors [61–63], etc.) and investigating their interactions with gallium may help optimize gallium nitrate as an antipseudomonal therapeutic.

## MATERIALS AND METHODS

### Bacterial strains and growth conditions.

P. aeruginosa strain PAO1, pyoverdine biosynthetic mutant (PAO1*ΔpvdF*), and pyochelin biosynthetic mutant (PAO1*ΔpchBA*) were provided by Dieter Haas. Deidentified P. aeruginosa isolates from pediatric cystic fibrosis patients were provided by Carolyn Cannon (7). For all experiments, P. aeruginosa was grown in modified M9 medium (1% 5× M9 salts [Difco], 3% low-iron Casamino Acids [Difco], 1 mM MgSO_4_, 1 mM CaCl_2_) after initial inoculation of 100-fold diluted overnight culture. Bacteria were grown in 96-well plates for 12 h at 37°C. Bacterial growth (absorbance at 600 nm) and pyoverdine production (excitation [Ex.], 405 nm; emission [Em.], 406 nm) were measured using a Cytation5 multimode plate reader (BioTek).

### P. aeruginosa whole-genome sequence analysis.

Bacterial genomic DNA was purified from overnight culture using DNeasy UltraClean Microbial kit (Qiagen). Paired-end Illumina sequencing was performed by the Microbial Genome Sequencing Center (MiGS) (Pittsburgh, PA) for at least 40× genome coverage. For the identification of aminoglycoside-modifying enzymes, raw sequences were first assembled via SPAdes (64) and annotated via Prokka (65). Mutation analysis in gallium-resistant mutants was performed using *breseq* (66).

### P. aeruginosa biofilm formation assay.

P. aeruginosa bacteria were grown in M9 medium after initial inoculation of 100-fold diluted overnight culture in 12-well plates (1 ml per well) at 30°C for 20 h. P. aeruginosa biofilms were stained with 1 ml crystal violet solution (0.1% crystal violet in 20% ethanol) for 30 min after aspirating the culture supernatant. After the biofilms were gently washed in S basal buffer twice, the stained biofilms were dried at 37°C. To quantify biofilm formation, the crystal violet stain was solubilized in 30% acetic acid, and absorbance at 550 nm was measured using a Cytation5 multimode plate reader (BioTek) (67).

### C. elegans pathogenesis assay.

C. elegans-P. aeruginosa liquid killing was performed as previously described (68). In brief, C. elegans nematodes were treated with P. aeruginosa in liquid kill medium (25% SK medium [0.3% NaCl, 0.35% Bacto peptone in water] in S basal buffer [100 mM NaCl, 50 mM potassium phosphate {pH 6.0}]) after initial inoculation of saturated overnight culture to an optical density at 600 nm (OD_600_) of 0.03 in 384-well plates. After 68 h of incubation at 25°C, all wells were extensively washed with S basal buffer and treated with Sytox Orange nucleic acid stain for 12 h (ThermoFisher Scientific) to label dead organisms. Bright-field and fluorescent images were acquired on a Cytation5 multimode plate reader (BioTek) and analyzed via Cell Profiler (www.cellprofiler.org) using a previously established pipeline (69).

### Drug interaction analysis.

Drug interactions and synergy scores (δ-scores) were calculated using SynergyFinder 2.0, an online tool based on the Bliss, highest single agency (HSA), and zero interaction potency (ZIP) synergy models (28).
